# Predicting attrition of men with a history of violence from randomised clinical trials

**DOI:** 10.1186/s13063-023-07774-3

**Published:** 2023-11-17

**Authors:** Natalie Doring, Ye In (Jane) Hwang, Emaediong Akpanekpo, Mathew Gullotta, Bianca Ton, Lee Knight, Crosbi Knight, Peter Schofield, Tony Gerard Butler

**Affiliations:** 1 School of Population Health, University of New South Wales, Sydney, NSW Australia; 2 The University of New Castle, Callaghan, NSW Australia; 3 Neuropsychiatry Service, Hunter New England Mental Health, Newcastle, NSW Australia

**Keywords:** Attrition, Retention, Randomised clinical trials, Treatment attrition, Offender intervention, Dropout

## Abstract

Preventing dropout (attrition) from clinical trials is vital for improving study validity. Dropout is particularly important in justice-involved populations as they can be very challenging to engage and recruit in the first instance. This study identifies factors associated with dropout in a double-blind, placebo-controlled randomised control trial of a selective serotonin reuptake inhibitor (SSRI) aimed at reducing reoffending in highly impulsive men with histories of violent offending. Age, education, social support, psychiatric history, and length of previous incarceration were identified as factors that predict attrition. These findings are consistent with previous research examining variables associated with attrition in clinical trials for community and offender populations. We also explored referral source and treatment allocation as attrition predictors. Although neither significantly predicted attrition, we identified that there are discernible differences in the median time to attrition among the referral source subgroups. Understanding factors that predict treatment completion and attrition will allow researchers to identify participants for whom additional provisions may optimise retention and inform development of targeted interventions.

**Supplementary Information:**

The online version contains supplementary material available at 10.1186/s13063-023-07774-3.

In 2016, 2.2 million adults in Australia were estimated to have been victims of domestic violence by age 15 [1]. For victims of violent crime, the impacts can be profound, including an increased risk of anxiety, depression, post-traumatic stress disorder, and substance use [2–4]. Additionally, there is a significant financial burden of such crime, with estimates suggesting the cost of assault, robbery, and homicide in Australia is $1800, $3600, and $1.6 million per incident, respectively [5] and the cost of domestic violence against women and children to be $20 billion per year [6]. Thus, given the health, social, and economic impacts of violence and domestic violence, research aimed at prevention is vital.

Randomised control trials (RCT) represent the gold standard in medical research [7]. They are posited as necessary to offender studies, particularly pharmacological clinical trials that demonstrate the effectiveness of interventions aimed at offender rehabilitation [8]. In light of this, it might be expected that RCTs are widely used to investigate crime-reduction interventions. However, such studies are relatively uncommon in this field [9, 10].

One reason proposed to account for the lack of RCTs with offenders is that they fall into a group dubbed “hard to treat” by many researchers [11]. This is due, in part, to ethical challenges of conducting research with offenders and their chaotic lifestyles characterised by itinerancy, insecure accommodation, lack of reliable contact methods (e.g. frequent changing of mobile phones or limited internet access), and turbulent social connections [12–14]. These factors make research completion particularly challenging, resulting in offender studies having higher rates of attrition (i.e. “1-loss to follow-up”; Brueton, 2014, p.14) [15] than non-offender studies [16]. Consequently, it has been suggested that aspiring to conduct RCTs in the justice system is too challenging, and lesser levels of evidence should be considered [17].

Typically, RCTs with offenders report that around half of the sample is lost to attrition. For example, Butler et al., Coccaro et al., Cullen et al., Kraanen et al., Lardén, and Stone et al. report an average attrition rate of 53% [18–23]. This is substantially higher than the average attrition rate of 35% reported in community health research [17, 24, 25]. One consequence of high attrition is the negative impact it can have on the validity of clinical trials [26, 27]. Perhaps even more pressing is evidence suggesting offenders who drop out of research interventions and trials are more likely to re-offend, suggesting a need for a better understanding of reasons for dropout from trials in the justice field [28].

In Australia, those who commit violent crimes such as homicide, assault, sexual assault, and robbery, tend to commit repeat offences [29, 30]. Forty-four percent of individuals convicted of assault and 47% convicted of robbery returned to prison within two years of release [31]. Maximising retention in programmes aimed at reducing reoffending via an improved understanding of factors that predict and affect attrition will be highly useful for reducing violence.

Efforts to predict attrition of violent offenders in clinical trials and treatment programmes have identified “attrition risk factors” among offenders convicted of domestic violence or sex crimes (e.g. sexual assault or child sex offences). These include a lack of social support; a history of alcohol abuse or poor mental health; being younger, unemployed, single, childless, expelled from school, or an ethnic minority; and having low incomes, little formal education, a learning disability, insecure accommodation, dysfunctional expressions of anger, or prior history of criminality [32–38]. Referral source has been proposed as a risk factor; however, there is a lack of consensus as to whether court-mandated diversion into treatment programmes protects against attrition or is a risk factor [39–41].

A limited number of studies have looked at predicting attrition from clinical trials and treatment programmes among individuals who commit violent crimes other than domestic violence and sex crimes (e.g. armed robbery, grievous bodily harm, manslaughter, malicious wounding, or assault). These studies show that impulsivity, depression, and indigenous heritage are associated with attrition [42–44]. To our knowledge, only two studies report treating violent populations with pharmacological means [18, 19]. While both of these studies experienced high treatment attrition (41% from Butler’s pilot study and 45% Coccaro’s study with individuals with impulsive aggressive behaviour) [19, 45] little consideration was given to describing factors associated with attrition. Coccaro et al. reported no difference between completers and non-completers regarding impulsivity and aggression scores [19]. Butler et al. suggested that attrition was related to the challenging nature of the client group (i.e. frequently changing phone numbers, transient living arrangements, poor time management, and impulsivity) [18]. Identifying factors that can predict attrition of violent offenders in pharmacological-based trials has significant implications, particularly given the promising results of both studies [19, 45].

This study aimed to identify factors that predict attrition from pharmacological-based treatments. Specifically, we investigated predictors of attrition in a double-blind, randomised, placebo-controlled trial (ReINVEST) by men with histories of repeat violent offending [18].

## Methods

### Data source

The study design, which has been extensively described in previous publications [18, 45], utilises data obtained from the ReINVEST clinical trial. The ReINVEST trial is a two-arm, parallel-group, double-blind, placebo-controlled RCT that was conducted to evaluate the effectiveness of sertraline (a selective serotonin reuptake inhibitor (SSRI)) in reducing reoffending rates among individuals with a history of violent offending.

### Ethics

This study has received ethical approval from the University of New South Wales (HC17771), Aboriginal Health & Medical Research Council (AHMRC; 822/11), Corrective Services NSW (09/26576) and the NSW Justice Health (G8/14) and the ReINVEST study is registered with the Australian and New Zealand Clinical Trials Registry (ACTRN12613000442707).

### Participants

The study population is comprised of 628 (*M*age = 32.25 years; *S.D* = 9.88) male participants enrolled in the ReINVEST clinical trial. The primary sources of referrals for participant recruitment in the study were Australian local magistrates’ courts, Legal Aid, New South Wales (NSW) solicitors, private lawyers, Corrective Services NSW Community Corrections Officers, and passive recruitment methods (i.e. flyers available at courts and other locations, word-of-mouth, self-referral, a free call number and study website) from October 2013 to June 2021. Medically fit males over the age of 18 with the ability to communicate in English, an impulsivity score (measured by the Barratt Impulsiveness Scale [46]) of 70 or more, two or more prior convictions for violent offences (excluding homicide or a child sexual offence), no serious mental illness (e.g. schizophrenia), and were not already on an SSRI were eligible for ReINVEST. Eligible participants were offered $50 for the initial screening visit, $20 per subsequent assessment (every 4 weeks), and $10 per visit when collecting medication to cover expenses incurred because of participation.

After screening, eligible participants then underwent a comprehensive psychiatric assessment and medical examination, administration of a number of behavioural measures, and collection of demographic data. A detailed list of all study assessments (including abbreviations) used during the recruitment phase of the ReINVEST clinical trial is available in Butler et al. (2021) [18]. Of these, this study used assessments of factors reported to predict attrition (Table 1). All participants completed a four-week run-in phase when all received the active medication prior to randomisation. During the run-in, all received a daily dose of 100 mg of sertraline taken orally. This was to identify those who react poorly to the medication or are not willing to commit to 12 months follow-up after initially consenting. Participants were then randomised into either the treatment or control arms of the study, with controls receiving an identical-looking placebo tablet. After study completion (12 months) all participants were offered the option to remain in the study or cease protocol treatment. A number of steps were taken to avoid potential sources of bias including employing objective, validated, standardised behavioural measures (Table 1), using an online randomisation system for treatment allocation, and used the STROBE checklist to enhance transparent reporting [47].

**Table 1:** Details of study assessments

Measure	Details
Demographic and criminographic information	Age, Aboriginal status, relationship status, accommodation, number of dependent children, level of education, requiring learning support in school, school expulsion, history of crime as a juvenile, history of violent juvenile crime, recent violent offences, incarceration history, referral source
Barratt Impulsiveness Scale (BIS) [46]	30-item questionnaire that assesses three subtypes of trait impulsiveness: attentional impulsiveness, motor impulsiveness and non-planning impulsiveness. Sound internal consistency and test–retest reliability (Cronbach’s *α* = 0.83 and Spearman’s rho = 0.83) [48, 49]
Duke Social Support Index (DSSI) [50]	11-item questionnaire that provides a validated index of the degree of social support available to the participant. Sound internal reliability and construct validity (Cronbach’s *α* = 0.80) [51]
Beck Depression Inventory Second Edition (BDI-II) [52]	21-item questionnaire that enquires about symptoms over the past week to measure the severity of depression. Strong internal and test–retest reliability (Cronbach’s *α* = 0.9; 0.73 to 0.96) [53]
Kessler Psychological Distress Scale (K10) [54]	10-item questionnaire intended to yield a global measure of distress based on questions about anxiety and depressive symptoms in the past 4 weeks. Sound convergent and criterion validity (0.87 to 0.88), sound internal consistency (Cronbach’s *α*= 0.89) [55, 56].
Anger, Irritability, and Assault Questionnaire (AIAQ) [57]	42-item questionnaire that records subjective levels of anger, irritability and aggression in the past 2 weeks. Sound validity and reliability (subscales coefficients range = .57 to .94) [57]
Alcohol Use Disorders Identification Scale (AUDIT) [58]	Measures alcohol consumption in the previous 12 months. Indicates safe, harmful and hazardous alcohol use Sound internal and test–retest reliability (Cronbach’s *α*= 0.85 to 0.92) [59, 60].

### Outcome and definitions

The primary outcome of interest in this study was time to attrition post-randomisation from the ReINVEST trial. The secondary outcomes were socio-demographic, justice-related and mental health factors associated with attrition from ReINVEST trial. Attrition was defined as the termination of a participant's involvement in the trial before its completion. Attrition was assessed based on several reasons, including the occurrence of adverse events, loss to follow-up (three consecutively missed appointments with no further contact), physical or mental health concerns, reincarceration, perceived lack of benefit, and personal choice.

For this study, the follow-up period was defined as the timeline from randomisation (baseline) until the earliest of the following: attrition, death, and completion of the study at 24 months post-randomisation. Although participants could cease protocol treatment at 12 months, 24 months was used to capture those participants who opted to remain in the study beyond 12 months.

### Covariates

The following covariates were included in the present analysis: randomisation status (main exposure), age, Aboriginal status, relationship status, accommodation type, number of children, highest educational level, requiring educational support during school, being expelled from school, history of juvenile violent offending, time spent in juvenile detention, number of violent offences within 5 years prior to randomisation, length of incarceration within 5 years prior to randomisation, psychiatric history, referral source, scores on the DSSI [50], BIS [46], BDI-II [52], K10 [54], AIAQ [57], and AUDIT [58].

### Statistical analysis

Descriptive statistics were used to summarise participant characteristics at randomisation (baseline). The time at risk was calculated as the duration from randomisation until the earliest of the following events: attrition, death, or 24 months post-randomisation. Kaplan–Meier method was used to estimate the cumulative incidence of attrition, overall and stratified by referral source. To identify factors associated with attrition, univariable and multivariable Cox regression models were fitted. Death was considered as a competing risk in this study. However, no participants died during the follow-up period. The multivariate model incorporated all covariates of interest.

The initial multivariate model incorporated all covariates with a *P*-value of < 0.2 on univariate analysis. Covariates with a *P*-value of < 0.05 were retained in the final model. Crude and adjusted hazard ratios (aHR) were derived along with their corresponding 95% confidence intervals (95% CI) as measures of association. To evaluate the underlying assumptions and goodness of fit of the Cox proportional hazards regression model, several diagnostic techniques were utilised. The proportional hazards assumption was assessed by analysing Schoenfeld residuals. The cumulative hazards based on Cox-Snell residuals were plotted against the Nelson-Aalen estimate of the cumulative hazard, and the unit slope was visually examined to assess model fit. All statistical analyses were conducted using a two-tailed significance level of 0.05. Data analysis was conducted using Stata version 17 (Stata Corporation, College Station, TX, USA).

## Results

The characteristics of the study population at baseline are summarised in Table 2.

**Table 2:** Characteristics of the study population: overall and stratified by attrition during the 24-month follow-up

**Characteristics**	**Overall** (*N* = 628)	**Dropped out before 24-month follow-up**	
		No (*n* = 237)	Yes (*n* = 391)
**Group**			
Placebo	309 (49.2)	113 (47.7)	196 (50.1)
Sertraline	318 (50.2)	124 (52.3)	195 (49.9)
**Age (years)**			
< 25	169 (26.9)	50 (21.1)	119 (26.9)
25–34	223 (35.5)	76 (32.1)	147 (37.6)
35–44	152 (24.2)	61 (25.7)	91 (23.3)
≥ 45	84 (13.4)	50 (21.1)	34 (8.7)
**Aboriginal status**			
Non-ATSI	444 (70.9)	181 (76.4)	263 (67.6)
ATSI	182 (29.1)	56 (23.6)	126 (32.4)
**Currently in a relationship**	252 (40.1)	90 (38.0)	162 (41.4)
**Accommodation status**			
Renting/government housing	445 (70.9)	162 (68.4)	283 (72.4)
Own home/living with family	135 (21.5)	56 (23.6)	79 (20.2)
Insecure accommodation	48 (7.6)	19 (8.0)	29 (7.4)
**Number of children, median (IQR)**	1 (0, 3)	1 (0, 3)	1 (0, 3)
**Education category**			
Did not complete school	215 (34.6)	75 (32.2)	140 (36.0)
School certificate	220 (35.4)	71 (30.5)	149 (38.3)
HSC/VCE/leaving certificate	63 (10.1)	30 (12.9)	3 (8.5)
Certificate/diploma/tech/trade	111 (17.9)	50 (21.5)	61 (15.7)
Degree/tertiary qualification	13 (2.1)	7 (3.0)	6 (1.5)
**Required educational support in school**^**b**^	188 (29.9)	73 (30.8)	115 (29.7)
**Expelled from school**^**b**^	235 (37.4)	98 (41.4)	137 (35.0)
**Juvenile offending history**			
No history of juvenile violent offending	562 (89.5)	214 (90.3)	348 (89.0)
History of juvenile violent offending	66 (10.5)	23 (9.7)	43 (11.0)
Time spent in juvenile detention for any offence	140 (22.3)	42 (17.7)	98 (25.1)
**Referral source**			
Community corrections	272 (43.3)	106 (44.7)	166 (42.5)
Magistrate/legal services	289 (46.0)	98 (41.4)	191 (48.9)
Self/other	67 (10.7)	33 (13.9)	34 (8.7)
**Psychiatric history**			
No admission/treatment/medication	249 (39.8)	83 (35.2)	166 (39.8)
≥ 1 treatment/medication (no admission)	277 (44.3)	113 (47.9)	164 (42.2)
≥ 1 psych admission	99 (15.8)	40 (16.9)	59 (15.2)
**Number of prior violent offences**^**a**^			
0	65 (10.4)	28 (11.8)	37 (10.4)
1–2	304 (48.4)	103 (43.5)	201 (51.4)
3–4	159 (25.3)	63 (26.6)	96 (24.6)
≥ 5	100 (15.9)	43 (18.1)	57 (14.6)
**Duration of prior incarceration**^**a**^			
0	326 (51.9)	127 (53.6)	199 (50.9)
1–6 months	188 (29.9)	75 (31.7)	113 (28.9)
7–12 months	63 (10.1)	22 (9.3)	41 (10.5)
> 1 year	51 (8.1)	13 (5.5)	38 (9.7)
**BIS Score, median (IQR)**	85 (77, 92)	85 (79, 92)	84 (77, 91)
**DSSI Score, median (IQR)**	25 (21, 28)	25 (22, 28)	25 (20, 28)
**BDI-II Score, median (IQR)**	9 (4, 15)	9 (3, 15)	9 (4, 15)
**K10 Score, median (IQR)**	14 (8, 21)	14 (8, 20)	14 (8, 22)
**AIAQ Score, median (IQR)**	71 (54, 87)	73 (55, 90)	70 (53, 86)
**AUDIT Score, median (IQR)**	10 (4, 17)	9 (3, 16)	10 (4, 17)

^a^Assessed within 5 years prior to randomisation. *BIS *Barratt Impulsiveness Scale [46], *DSSI *Duke Social Support Scale [50], *BDI-II *Beck Depression Inventory Second Edition [52], *K10 *Kessler Psychological Distress Scale [54], *AIAQ *Anger, Irritability, and Assault Questionnaire [57], *AUDIT* Alcohol Use Disorders Identification Scale [58]

^b^Missing category not included. *Note*: Current relationship = married/de facto

**Fig. 1:**
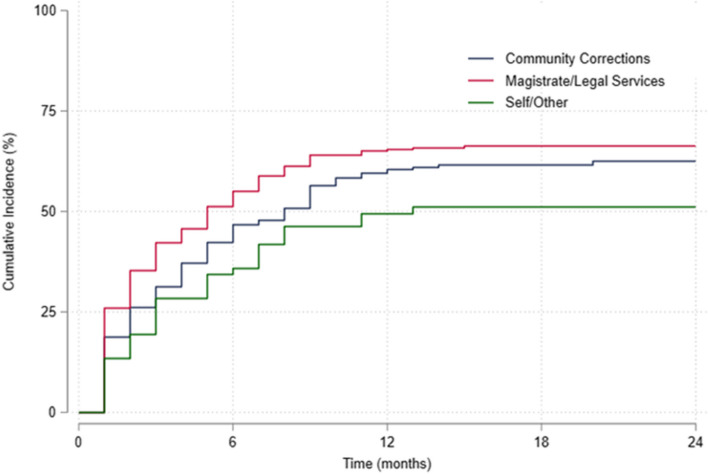
Estimated cumulative incidence of attrition from ReINVEST, stratified by referral source (*N* = 628)

Given the interest in the source of referral as an attrition predictor [39, 40], we also assessed the association between referral source and attrition. Those who were referred by a magistrate/legal services tended to exit the study earlier compared to other referral sources (Fig. 1). Cumulative incidence of dropout at 24 months post-randomisation was 50.6% for self/others referral, 62.5% for community corrections referral, and 65.6% for magistrate/legal services referral. The median time to attrition is 11 months (IQR: 3, 19) for self/others, 8 months (IQR: 2, 13) for community corrections and 5 months (IQR: 1, 14) for magistrate/legal services.

Loss to follow-up was the main reason for attrition overall (56.3%) but the proportion was higher in those referred by magistrates/legal services (63.9%) compared to self/other referred (58.8%) and community corrections referred (47.0%). Offence-related reasons for attrition overall were 9.7%, magistrate/legal service referred (5.8%), self/other referred (11.8%) and community corrections referred (13.9%). Unwanted side effects accounted for the least numbers in terms of attrition overall (3.3%), magistrate/legal services referred (4.7%), self-referred (0%) and community corrections referred (2.4%).

The multivariable analysis identified the following predictors associated with attrition (Table 3): age, educational attainment, incarceration history, psychiatric history, and DSSI score.

**Table 3:** Factors associated with participant attrition from ReINVEST (*n* = 628)

Characteristic	Attrition/person-years	Univariate analysis		Multivariate analysis	
		**Unadjusted HR** **(95% CI)**	***P***	**Adjusted HR** **(95% CI)**	***P***
**Group**			0.63		0.50
Placebo	196/228	1.0 (*ref*)		1.0 (*ref*)	
Sertraline	195/245	0.95 (0.78–1.16)		0.93 (0.75–1.15)	
**Age (years)**			< 0.01		< 0.01
	-	0.98 (0.96–0.98)		0.97 (0.96–0.98)	
**Aboriginal status**			0.03		0.13
Non-ATSI	263/344	1.0 (*ref*)		1.0 (*ref*)	
ATSI	126/128	1.25 (1.01–1.55)		1.21 (0.95–1.55)	
**Relationship status**			0.85		0.84
No current relationship	229/282	1.0 (*ref*)		1.0 (*ref*)	
Current relationship (married/de facto)	162/191	1.02 (0.83–1.25)		1.02 (0.81–1.29)	
**Accommodation status**			0.44		0.83
Renting/government housing	283/325	1.0 (*ref*)		1.0 (*ref*)	
Own home/living with family	79/112	0.85 (0.66–1.09)		0.93 (0.71–1.22)	
Insecure accommodation	28/35	0.92 (0.63–1.36)		1.05 (0.69–1.59)	
**Number of children**^**a**^			0.22		0.79
	-	0.97 (0.93–1.02)		0.99 (0.94–1.05)	
**Education category**			0.05		0.03
Did not complete School	140/159	1.0 (*ref*)		1.0 (*ref*)	
School certificate	149/146	1.08 (0.86–1.36)		1.01 (0.78–1.29)	
HSC/VCE/leaving certificate	33/55	0.72 (0.49–1.05)		0.58 (0.38–0.89)	
Certificate, diploma, tech/trade	61/98	0.78 (0.58–1.06)		0.79 (0.57–1.10)	
Degree/tertiary qualification	6/11	0.60 (0.26–1.36)		0.44 (0.16–1.22)	
**Educational support required**^**c**^			0.37		0.26
No	274/322	1.0 (*ref*)		1.0 (*ref*)	
Yes	14/147	0.91 (0.73–1.14)		0.87 (0.68–1.10)	
**Expelled from school**^**c**^			0.14		0.09
No	253/288	1.0 (*ref*)		1.0 (*ref*)	
Yes	135/180	0.86 (0.69–1.06)		0.81 (0.63–1.03)	
**Juvenile offending**			0.11		0.31
No history of juvenile violent offending	293/380	1.0 (*ref*)		1.0 (*ref*)	
History of juvenile violent offending	42/43	1.17 (0.84–1.61)		1.07 (0.74–1.55)	
History of time spent in juvenile detention	56/49	1.34 (1.00–1.78)		1.29 (0.93–1.79)	
**Referral category**			0.04		0.36
Community corrections	166/205	1.0 (*ref*)		1.0 (*ref*)	
Magistrate/legal services	191/204	1.17 (0.96–1.45)		1.14 (0.89–1.47)	
Self/other	34/62	0.76 (0.53–1.10)		0.89 (0.60–1.33)	
**Number of previous violent offences**^**ab**^			0.14		0.12
	-	0.97 (0.92–1.01)		0.91 (0.80–1.02)	
**Previous incarceration**^**b**^			0.03		0.03
≤ 1 year	353/445	1.0 (*ref*)		1.0 (*ref*)	
> 1 year	38/28	1.46 (1.04–2.04)		1.56 (1.05–2.31)	
**Psychiatric history**			0.03		0.03
No admission/treatment/medication	166/169	1.0 (*ref*)		1.0 (*ref*)	
≥ 1 treatment/medication/admission	223/302	0.79 (0.65–0.97)		0.78 (0.63–0.97)	
**BIS Score**^**a**^			0.16		0.51
	-	0.96 (0.92–1.01)		0.98 (0.92–1.04)	
**AIAQ Score**^**a**^			0.18		0.23
	-	0.98 (0.97–1.00)		0.98 (0.96–1.01)	
**AUDIT Score**^**a**^			0.53		0.70
	-	1.02 (0.96–1.08)		1.01 (0.95–1.08)	
**DSSI Score**^**a**^			0.07		0.03
	-	0.90 (0.81–1.00)		0.86 (0.75–0.98)	
**K-10 Score**^**a**^			0.52		0.73
	-	1.02 (0.96–1.08)		0.98 (0.91–1.07)	
**BDI Score**^**a**^			0.68		0.38
	-	1.01 (0.96–1.07)		1.04 (0.96–1.12)	

*HR* hazards ratio, *95% CI* 95% confidence interval. Global *p*-values are tests for heterogeneity excluding missing values

^a^Age, number of children, number of previous offences, BIS Score, AIAQ Score, AUDIT Score, Duke Score, Kessler-10 Score, and BDI Score assessed as continuous variables

^b^Number of violent offences and previous incarcerations assessed within 5 years prior to randomisation

^c^Missing category not included. *Note*: No current relationship = single/separated/divorced/widowed

Being older, higher levels of education, and higher levels of social support serve as protective factors against attrition (Table 3). A psychiatric history requiring medication or hospital admission reduced the risk of dropout, and a smaller cumulative duration of incarceration is associated with dropout.

## Discussion

The current study explored factors associated with attrition from a clinical trial involving pharmacotherapy aimed at treating impulsive individuals with a history of violent offending. Specifically, we investigated whether attrition from the ReINVEST trial could be predicted by demographic and psychosocial factors identified as attrition risk factors from the literature. Our results found that older age, higher levels of social support, higher levels of education, and psychiatric history were all protective factors against attrition at 24 months, whereas less time spent incarcerated in the past 5 years is associated with dropout. We found no association between referral source or treatment allocation on attrition.

Our findings that older age is associated with decreased attrition at 24 months are in line with past studies [61–63]. Sampson and Laub’s [64] (p.37) revised age-graded theory proposes that older offenders’ ability to display “purposeful execution of choice” is essential in criminal desistance. This purposeful choice execution may also explain older adults’ increased likelihood to remain in a trial perceived as being helpful in desistence. Neuroimaging studies show that older adults, compared to younger counterparts, perform better on decision-making tasks when previous choices influence an outcome or reward [65]. According to Grossman et al. older people are better at making decisions requiring higher-order processing of relational dependencies between recent choices and the available rewards [66]. Concerning retaining offenders in trials and programmes, older participants may be more likely to consider the consequences of previous anti/pro-social choices when deciding whether to drop out. Tailoring retention methods to account for the impact of age on decision-making may support retention more effectively. The NSW Behavioural Insights Unit (BIU) [67] (2018, p.15), recommends adopting a “bespoke approach adapted for offenders” that takes age into consideration. For those under 30 years, they recommend making contact early and emphasising benefits, such as programme completion being regarded in upcoming sentencing. For older offenders, who often report being “weary of prison, but are equally distrustful of programs” (BIU, 2018, p.15) they suggest offering clear information on how the trial can offer hope and help them reclaim a sense of control over their life. Qualitative research with participants and research staff exploring how age-dependant difference in decision-making impacts attrition from clinical trials is recommended.

Our finding that increased social support is protective against attrition is also in line with past research [68]. One explanation is that social support alleviates the impact of other attrition risk factors (e.g. poor mental health) [69, 70]. For example, being able to depend on others for emotional support reduces depressive symptoms, which have been linked to attrition [71]. Past research has linked attrition to the number of supports offenders have [72]. However, it is likely the quality, rather than the number, of social support that is important [73]. Socioemotional Selectivity Theory suggests that social network satisfaction decreases adverse lifestyle fluctuations [74], likely enhancing the stability needed to remain in trials and programmes.

Adopting a holistic approach when designing RCTs that foster quality in participants' social networks would enhance study retention. For example, employing case management or peer support strategies for those who report low social support at baseline may minimise dropout and enhance successful rehabilitation. Klaehn et al. (2022, p.294) [75] have demonstrated that case management is a promising “cost-effective, or even cost-saving” method to support retention of participants with complex needs, such as offenders. Many offenders are under judicial supervision (e.g. probation officers or Community Safety Case Managers). Collaboration between existing supervision providers and research teams offers the optimal chance to meet the shared goal of rehabilitation. It also offers justice-health researchers a unique opportunity to harness existing case-management networks. Further research exploring the impact of case-management collaboration and peer support strategies on the retention of people participating in justice system trials and programmes is suggested.

Our finding that lower levels of education are associated with increased attrition is consistent with other research [76–83]. There are several possible explanations why education is related to attrition. For example, higher levels of education are associated with increased cognitive function in areas such as attention, memory, and problem-solving skills [84] and enhanced ability to manage complex tasks [85]. It may be that individuals with higher levels of education are better able to manage the requirements of participation, whereas lower levels of education may be associated with the perceived complexity of the trial procedures, language, and concepts, leading to feelings of being overwhelmed. This sense of overwhelm, known as cognitive overload, may be one factor that drives the relationship between low education and attrition. Continued investigation into the relationship between cognitive overload and attrition may help elucidate this relationship further. Our findings suggest that providing additional support to participants who report lower levels of education at baseline may be an approach to minimising attrition.

We also found that participants who had spent less time incarcerated in the 5 years prior to randomisation had an increased rate of attrition. While this finding contradicts Zanis et al.’s (2009) [86] report that offenders whose most recent incarceration was longer were less likely to complete treatment, it is worth noting that their study was conducted with offenders completing community-based drug treatment rather than community offenders aiming to reduce impulsive violent behaviour. Since attrition is the result of an interaction between a programme and its participants [87], the lack of consistency of this predictor of attrition is not entirely surprising. It does, however, emphasise that a “one size fits all” approach should be avoided when attempting to reduce offender attrition. Whether incarceration length serves as a predictor of dropout may differ depending on the nature of offending behaviour or programme management and delivery.

Zapryanavo’s (2020) [86] report that each month spent incarcerated results in a 1.12 percentage decrease in the probability of reoffending while on parole offers context for our findings on incarceration and attrition. It may be that more lengthy experiences of incarceration leave participants determined to avoid recidivism; they perceive trial participation as a valuable way to avoid future incarceration. In contrast, individuals who have spent less time incarcerated may perceive less need for intervention, leading to early dropout. Alternatively, it could be that those who have spent less time in prison have more opportunities to become engaged in community activities such as employment, education, or family commitments, limiting available time to be involved in the trial.

Our findings also indicate that those with a history of psychiatric conditions requiring treatment, medication, or hospital admission are significantly less likely to drop out. This is contrary to past research suggesting that psychiatric disorders predict the attrition of violent offenders from programmes [87, 88]. Hoverer, those studies were reporting on dropout from behavioural interventions employing cognitive-behaviour therapy conducted in a group setting, rather than a clinical trial. Dishion et al. (1999) [89] report some individuals show increases in criminality after group interventions, possibly because of peer reinforcement of criminal thinking and behaviour. Reinforcement processes may also underlie attrition from group therapy. In contrast, being involved in a pharmacological trial delivered one-on-one with research staff offers participants access to mental health clinicians that they might not otherwise have access to, maintaining privacy and confidentiality, and avoiding reinforcement of undesirable behaviours. Additionally, participating in a clinical trial such as ReINVEST might be viewed as less stigmatising, less confronting, and less anxiety provoking then attending group-based behaviour therapy.

Our finding that referral source does not predict attrition is consistent with past studies that also report a null effect regarding referral source [90–92] and contradicts reports that court referral supports retention [93, 94]. It is often suggested that participation in trials like ReINVEST, which had a policy developed enabling referral from the bench by a magistrate, is motivated by a simple desire to avoid prison [95]. The fact that we saw the lowest proportion of attrition among those who were self-referred and over half (51%) of those enrolled were serving community sentences and not facing the prospect of prison suggests motivation for participation is more than a simple desire to avoid prison. It is also patronising to suggest that this is the prime and only motivation of this group. Indeed, a pre-study survey [96] of prisoner's potential willingness to join a trial such as ReINVEST found that over half the respondents were interested in learning more about the study, and of those respondents, 80% said they would still take part despite a 50% chance of receiving a placebo. This suggests that these offenders have insight into the negative impact of their anti-social behaviour, and they are interested and willing to be involved in treatment to reduce problematic violence.

If a relationship between referral source and attrition exists, it is possible the relationship is mediated by other factors. For example, research on why offenders dropout of psychological treatments indicates that younger, less educated men are more likely to remain in treatment if they are court mandated. In contrast, older, better-educated men may be more likely to remain in treatment if they are not court-ordered [97]. Further investigations into an interaction between referral source and ReINVEST attrition variables may help clarify whether a referral source and attrition relationship moderated by age and education in psychological treatments also exist in clinical trials.

Interestingly, we did not see an effect of treatment allocation on attrition. While this finding is consistent with Coccaro [19] who also report no effect of treatment condition on attrition from their fluoxetine/placebo trial, it contradicts reports of past RCTs that note attrition differences among treatment allocation groups [97–99]. However, these studies describe attrition from prison-based behaviour therapies (e.g. yoga, cognitive behaviour therapy, and exercise) rather than pharmacotherapy trials. Olver et al. [63] report attrition is impacted by treatment modality, with the highest attrition observed in behavioural therapy programmes. Our findings, and those of Coccaro [19] call to question suggestions that offering a placebo can impact attrition by introducing uncertainty sufficient to decrease the magnitude of response or motivation to remain engaged [99, 100].

It is possible that the unique nature of ReINVEST as a community-based trial that offered free access to follow-up appointments with research clinicians and a psychiatrist influenced retention, regardless of treatment allocation. It allowed participants, irrespective of their treatment allocation, to receive ongoing professional healthcare attention and support. This could be appealing, given services can be difficult to access and costly outside of the trial setting. Further, regular appointments provided an opportunity for participants to discuss any concerns or adverse effects, and to feel heard and validated. This sense of support may have enhanced motivation to remain in the trial regardless of treatment allocation. It is possible that for control participants regular appointments with clinicians and a psychiatrist reinforced any experienced placebo effect. The positive psychosocial benefits of attention and care offered as part of an effective treatment team may have been perceived as an improvement in their condition. This suggests that providing offenders access to frequent, consistent, high-quality healthcare can support retention in trials. Further, offenders may be more likely to remain committed to the study, regardless of treatment allocation, if offered empathetic communication that ensures they feel observed and cared for.

Previous research indicated that Aboriginal status, relationship status, number of dependents, accommodation type, impulsivity, anger, irritability, and alcohol abuse predict offender dropout from research interventions and clinical trials [63, 101, 102]. Despite this, we found no support for these factors as predictors of attrition in the ReINVEST trial. Null findings in a study exploring attrition can be beneficial information to have. It may indicate that researchers conducting pharmacological trials do not need to be overly concerned that these participant characteristics will bias outcomes. However, the unique nature of our sample (all were highly impulsive men with histories of violence) may explain why our findings differ from past studies.

## Limitations

A limitation of this study is it is based on a sample of highly impulsive repeat-violent offenders. Additionally, reliance on self-report to ascertain the impact of some of the variables on attrition (e.g. relationship status, number of dependents, accommodation type, impulsivity, anger, irritability, and alcohol abuse) may be subject to recall or response bias. It is possible that individuals who have a history of committing violent offences may feel the need to manage their public identity and may have modified their responses. Notwithstanding these limitations, this study also possesses considerable strengths. We have identified age, social support, education, time spent incarcerated, and psychiatric history are associated with attrition.

## Conclusions

The problem of offender attrition from clinical trials and programmes is ongoing and a serious concern given it is linked to recidivism. Identifying ways to reduce the attrition from clinical trials of offenders who commit violent crimes is vital. This study identified a number of risk factors that support researchers to predict attrition of men with a history of violence from clinical trials. Awareness of these factors can support researchers working with samples that are justice-involved to determine, at baseline, participants for whom additional provisions may optimise retention.

### Supplementary Information

**Additional file 1.**

## Data Availability

Records management is guided by systems implemented by the NHMRC Clinical Trials Centre, in line with requirements for record keeping in the NSW private sector, medico-legal requirements and the NSW State Records Act 1998. Trial documents are stored under medical records conditions for 15 years post publication in the secure UNSW Shared File storage, with access restricted to the appropriate researchers who have access to UNSWnetworks. On the case report forms and other documents, a unique study code is used to identify the records. The datasets used and/or analysed during the current study are available from the corresponding author on reasonable request.
